# Visualizing the Nucleotide Addition Cycle of Viral RNA-Dependent RNA Polymerase

**DOI:** 10.3390/v10010024

**Published:** 2018-01-04

**Authors:** Jiqin Wu, Peng Gong

**Affiliations:** 1 Key Laboratory of Special Pathogens and Biosafety, Wuhan Institute of Virology, Chinese Academy of Sciences, Wuhan 430071, China; wujiqin2010@163.com; 2 University of Chinese Academy of Sciences, Beijing 100049, China

**Keywords:** RNA viruses, RNA-dependent RNA polymerase, nucleotide addition cycle, active site closure, translocation

## Abstract

Viral RNA-dependent RNA polymerases (RdRPs) are a class of nucleic acid polymerases bearing unique features from global architecture to catalytic mechanisms. In recent years, numerous viral RdRP crystal structures have improved the understanding of these molecular machines, in particular, for how they carry out each nucleotide addition cycle (NAC) as directed by the RNA template. This review focuses on a visual introduction of viral RdRP NAC mechanisms through a combination of static pictures of structural models, a user-friendly software-based assembly of the structural models, and two videos illustrating key conformational changes in the NAC.

## 1. Introduction

The RNA-dependent RNA polymerases (RdRPs) encoded by RNA viruses share a unique encircled right-hand catalytic core of about 50–70 kD with seven catalytic motifs A–G surrounding the active site in a structurally conserved manner [1,2,3,4,5,6,7]. All other right-hand-shape polymerases, represented by the *Escherichia coli* DNA polymerase I (*E. coli* DNAP I), bacteriophage T7 RNA polymerase (T7 RNAP), and human immunodeficiency virus 1 (HIV-1) reverse transcriptase (RT), do not have the encircled architecture [8,9,10,11]. Structurally, RTs share motifs A–F with RdRPs but do not have motif G equivalents, while *E. coli* DNAP I, T7 RNAP, and their relatives primarily share motifs A and C with RdRPs, with each motif containing a universally conserved aspartic acid residue [12]. The characteristics of the RdRP structure contribute to the uniqueness of their catalytic mechanisms, particularly with respect to the nucleotide addition cycle (NAC). The NAC is the central content of polymerase catalysis, and each cycle primarily includes four sequential steps: nucleotide triphosphate (NTP) binding, active site closure, phosphodiester bond formation, and translocation [13]. Typically, the pre-chemistry active site closure and the post-chemistry translocation are accompanied by polymerase conformational changes and therefore are of great interest for understanding the nature of the NAC and for developing NAC-targeting antiviral strategies. In recent years, crystallographic data characterizing enterovirus RdRP elongation complexes (ECs) have provided a nearly complete structural view of the NAC in these RdRPs [12,14,15]. Together with structural data obtained in RdRPs from norovirus (NV), hepatitis C virus (HCV), foot-and-mouth disease virus (FMDV), and so forth, the key structural features of the NAC have been readily unraveled [16,17,18,19]. In this short review, we intend to sketch a visual landscape of the viral RdRP NAC by threading reference NAC states derived from available crystal structures. With the aid of the structure visualization program PyMOL (The PyMOL Molecular Graphics System, Schrödinger, LLC) and videos illustrating key conformational changes, we seek to introduce the uniqueness of the viral RdRP NAC to a broader readership including researchers who are not familiar with three-dimensional structure visualization. For a mechanistic introduction of the uniqueness of viral RdRP NAC from a structural aspect, we refer to a recent review explicitly comparing the NAC of viral RdRPs and T7 RNAP [11].

## 2. Visualizing the Viral RNA-Dependent RNA Polymerases Nucleotide Addition Cycle States and Related Key Conformational Changes

**Figure 1:**
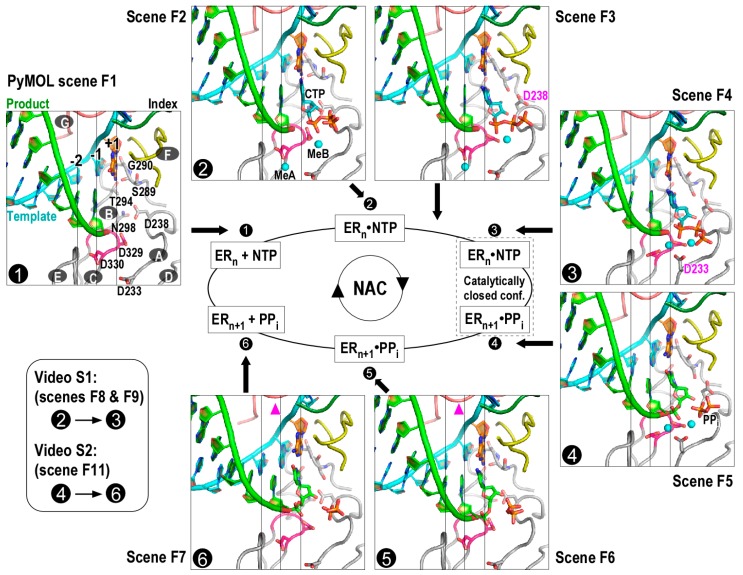
Viral RNA-dependent RNA polymerase nucleotide addition cycle (RdRP NAC) states. Seven RdRP EC models represent NAC reference states 1–6 and an intermediate state between states 2 and 3. Each model corresponds to a Scene in the PyMOL assembly (Supplementary file 1) that can be conveniently recalled in the PyMOL interface. Seven RdRP motifs A–G are labeled in the state 1 complex. Motif A contains two conserved aspartic acid residues (D233 and D238 in enterovirus 71 (EV71) RdRP) and undergoes backbone rearrangement during active site closure; motif B contains four conserved residues (S289, G290, T294, and N298), interacts with the nascent base pair (corresponding to position +1), and participates in NTP recognition and/or translocation; motif C contains the XXDD (the X residues are variable between viral families or genera and two Ds correspond to D329 and D330 in EV71 RdRP) sequence and participates in metal ion coordination and NTP binding; motif D is structurally associated with motif A and undergoes backbone movement during active site closure; motif E interacts with the product strand at the junction of −2 and −1 positions; motif F contains three conserved residues (K159, E161, and R174 in EV71 RdRP) and interacts with the +1 templating nucleotide and the NTP triphosphate moiety (key side chains and part of motif F are not shown for clarity); motif G runs approximately parallel to the template strand and contains two small-side-chain residues interacting with the +1/+2 junction of the template strand. Coloring scheme: template in cyan (+1 nucleotide in orange), product in green, palm in grey (YGDD sequence in magenta), index finger in dark green, ring finger (motif F) in yellow, and pinky finger (motif G) in pink. White numbers with black background indicate reference NAC states. White capital letters with dark grey background indicate RdRP motifs. Key side chains, the +1 template nucleotide, CTP, and pyrophosphate (PP_i_) are shown in sticks. Magnesium ions are shown as cyan spheres. Magenta triangles in the bottom panel indicate subtle movement of motif G in early stages of translocation. ER: enzyme-RNA complex.

Viral RdRPs begin their NAC with the state 1 complex (Figure 1, only structure in the left panel) with a vacant active site [12,14,15]. Subsequently, NTP binds in the active site and forms the state 2 complex (Figure 1, left structure in the top panel), and the polymerase conformation remains open at least in the initial binding stage [12,15]. In most cases, the binding of correct NTP induces conformational changes (also known as active site closure), leading to the formation of the catalytic competent state 3 complex (Figure 1, top structure in the right panel) [12,15,16,17]. The conformational changes primarily involve RdRP catalytic motifs A, D, B, and F. Among these motifs, only motifs A and D undergo notable backbone movements. They form antiparallel β-type interactions and move coordinately during active site closure and reopening. Two aspartic acid residues within motif A, a serine-glycine sequence in motif B, and one arginine residue in motif F undergo local but essential conformational changes during active site closure. Two catalytic metal ions, naturally Mg^2+^, are critical for the formation of the transition state of the phosphoryl transfer reaction [20]. The post-chemistry state 4 complex (Figure 1, bottom structure in the right panel) maintains the closed conformation active site [12,15]. Two events need to occur prior to the beginning of the next NAC: active site reopening and translocation. Largely on the basis of the observation of a pre-translocation structure with an open active site conformation obtained using deoxy NTP substrates, an RdRP-specific state 5 was proposed (Figure 1, right structure of the bottom panel) [12]. More recently, a very interesting translocation intermediate was captured by crystallographic methods and was assigned as the state 6 complex (Figure 1, left structure in the bottom panel) [15]. This structure suggests that the template-product RNA duplex may undergo asymmetric movement during early stages of translocation, with the product strand leading the template strand and the upstream portion leading the downstream portion. The structure also suggests that the interactions between the RdRP motif G and the −2 to +1 region of the template strand need to be disrupted and then reestablished to achieve the post translocation state (i.e., state 1 of the next NAC).

### 2.1. Model Generation of Reference Nucleotide Addition Cycle States

For illustration purpose, here we use seven RdRP EC models to represent the aforementioned six NAC states and an intermediate state between states 2 and 3 (Figure 1 and Table 1). These models were assembled into a PyMOL Session file for interactive visualization (Supplementary File S1). In order to provide convenient comparisons between the models, we chose an appropriate EV71 RdRP EC structure as the principal part of a particular model, and incorporated parts from appropriate poliovirus (PV)/NV RdRP structures to create a hybrid model where necessary. The details are listed as follows.

State 1 (PyMOL scene F1): An enterovirus 71 (EV71) RdRP EC model (Protein Data Bank (PDB) entry 5F8G) [15].

State 2 (PyMOL scene F2): This model was generated using an EV71 RdRP EC structure and a poliovirus (PV) RdRP EC structure. The RdRP and RNA were taken from the EV71 structure (PDB entry 5F8F) [15] and the cytidine triphosphate (CTP) was modeled on the basis of the 2′,3′-dideoxy-CTP (ddCTP) in a PV structure (PDB entry 3OLB) [12].

State 2/3 (PyMOL scene F3): An EV71 RdRP EC model (PDB entry 5F8I) that adopts a partially closed active site conformation [15]. Explicitly, the conformational change is around the NTP ribose, mainly involving motif A residue D238 and motif B residue S289.

State 3 (PyMOL scene F4): This model was generated using an EV71 RdRP EC structure and a NV RdRP–RNA–CTP complex structure. The RdRP and RNA were taken from the EV71 structure with the +1 product nucleotide removed (PDB entry 5F8J) [15], and the CTP was taken from the NV structure (PDB entry 3BSO) [16]. The EV71 structure was chosen because its RdRP active site adopts a catalytically closed conformation suitable for both states 3 and 4. The usage of the NV structure for CTP in the active site is due to the fact that it best demonstrates the situation of a closed conformation active site in the pre-chemistry stage. This NV structure itself is included in the PyMOL session file for comparison (Supplementary file 1, state nvS3).

State 4 (PyMOL scene F5): An EV71 RdRP EC model (PDB entry 5F8M) [15].

State 5 (PyMOL scene F6): This model was generated using an EV71 RdRP EC structure and a PV RdRP EC structure. The RdRP and RNA were taken from the EV71 structure with the −1 product nucleotide removed (PDB entry 5F8G) [15], and the −1 and +1 product nucleotides were modeled on the basis of the PV structure (PDB entry 3OL9) that contains a 3′-deoxy-CMP (3dCMP) at position +1 [12].

State 6 (PyMOL scene F7): An EV71 RdRP EC model (PDB entry 5F8N) [15] that adopts an intermediate conformation between the pre- and post-translocation states.

### 2.2. Generation of Videos Illustrating Key Conformation Changes

Two videos were generated for the visualization of key conformational changes yet identified in viral RdRP NAC.

Video 1 (PyMOL scenes F8 and F9, Supplementary Video S1): This movie illustrates the conformational changes related to active site closure. It connects states 2 and 3 through the intermediate state 2/3. Key distances (dist01-dist05 for connection between state 2 and state 2/3 and dist06-dist10 for connection between state 2/3 and state 3 in Supplementary file 1) are indicated by dashed lines to facilitate the visualization of the maintenance of the base-paring interactions and the establishment of a hydrogen bonding network around the NTP ribose 2′-hydroxyl group.

Video 2 (PyMOL scene F11, Supplementary Video S2): This movie illustrates the conformational changes in the early stages of translocation. For simplicity, this movie connects states 4 and 6. As a consequence, translocation in this movie is accompanied by the active site reopening. However, these two events are probably not tightly coupled as previously suggested [11,15].

Each video was generated by the “morph” command in PyMOL. Given the starting and ending states, an interpolated trajectory was created, and each intermediate state was refined to clean distortions.

## 3. Correlation between Structural and Biochemical Data

Biochemical data characterizing enzyme kinetics of viral RdRP NAC provide important information complementing the structural data. A symmetrical primer/template substrate (sym/sub) was used in the assembly of PV RdRP EC and to solve a relatively complete kinetic mechanism of NAC in a uridine-directed ATP incorporation [21,22,23]. Somewhat similarly to the six-state structural model, each NAC contains five biochemically defined steps. The first step is the NTP binding and corresponds to the structural switching from state 1 to state 2 (ER_n_ to ER_n_•NTP). The second step is partially rate-limiting and is associated with a conformational change (termed “isomerization” in these studies) leading to the catalytically competent state [22] and therefore likely matching the active site closure defined by the structural switching from state 2 to state 3 ([ER_n_•NTP]_open_ to [ER_n_•NTP]_closed_). The third step is the phosphoryl transfer and was also partially rate limiting ([ER_n_•NTP]_closed_ to [ER_n+1_•PP_i_]_closed_) [21]. When Mg^2+^ was replaced by Mn^2+^, the phosphoryl transfer became the only rate-limiting step in the NAC [22]. The fourth step was also associated with a conformational change and with PP_i_ bound ([ER_n+1_•PP_i_]_closed_ to [ER_n+1_•PPi]_open_). This step likely corresponds to the active site reopening, denoted by the structural switching from state 4 to state 5. The fifth step is the release of PP_i_ (ER_n+1_•PP_i_ to ER_n+1_), possibly accompanying translocation and leading to the start of the next NAC.

At least two mechanistic details are not fully clarified with both the biochemical and structural data considered. Firstly, the biochemically identified isomerization step could either correspond to the entire active site closure (state 2 to state 3) or to the final rearrangement to reach the catalytically competent conformation (state 2/3 to state 3). The crystallographic observation of the state 2/3 structure suggests that the conformational changes for active site closure are initiated around motif D residue D238 (EV71) near the NTP ribose 2′-hydroxyl and then are propagated to motif D residue D233 (EV71) near the catalytic metal binding sites. Secondly, the post-chemistry isomerization could correspond either to the active site reopening or to translocation. As suggested in the biochemical study, the post-chemistry isomerization is nearly irreversible (with an equilibrium constant of 2.0 × 10^5^ and a reverse reaction rate constant of 2.5 × 10^−3^ s^−1^) [21]. The structural data of the EV71 RdRP EC including the translocation intermediate structure rather suggests that at least the relative movement of RNA to RdRP in the early stage of translocation is likely reversible [15]. Hence, the correlation of the biochemically-identified isomerization with active site reopening but not translocation is favored.

## 4. Perspectives

As described above, the current structural data have provided a detailed visual illustration of the majority of critical steps in a viral RdRP NAC. Together with biochemical data sketching a relatively complete NAC kinetic scheme, the nature of the NAC has been readily unraveled. Unknown conformational changes may still exist, in particular, in the late stages of translocation upon relative movement of the template strand RNA and the RdRP and with global rearrangement of the RdRP structure or rearrangement involving inter-RdRP cooperation, which have not yet been identified in the RdRP catalytic complex structures. Both motif B and motif G have been suggested to be involved in this step [15,24]. More specifically, the motif B loop of PV RdRP (residues 288–292) was found to adopt an “in” conformation consistent with the majority of other RdRPs and an interesting “out” conformation that, if put in the context of a catalytic complex, could facilitate the movement of the template strand during translocation [24], while two motif G residues (residues 114–115) may serve as a ratchet paw to catch and release the backbone of the template strand during each NAC [15]. The subtle movement of motif G is evident even in the early stages of translocation by comparing reference states 5 and 6 (indicated by the pink triangles in Figure 1). Further evidence, obtained by crystallography, enzymology, and molecular dynamics simulation, are likely necessary to address this important missing link in the viral RdRP NAC.

## Figures and Tables

**Table 1:** Key Protein Data Bank (PDB) entries used in generating structural models representing different viral RNA-dependent RNA polymerase nucleotide addition cycle (RdRP NAC) states and a list of related PDB entries.

NAC State	PDB(s) for Model Construction	Reference PDB(s)	Active Site Conformation
1	5F8G (EV71)	3OL6, 4K4S, 4K4T, 4K4U, 4K4V, 4K4W, 4K4X, 4K4Z, 4K50, 5F8G, 5F8L [12,14,15]	Open
2	5F8H (EV71), 3OLB (PV) ^a^	3OLA (chains I/M), 3OLB, 4K4Y, 5F8H [12,15]	Open
2/3	5F8I (EV71)	5F8I [15]	Partially closed
3	5F8J (EV71), 3BSO (NV) ^b^	3BSO [16]	Closed
4	5F8M (EV71)	3OL7, 3OL8, 5F8J, 5F8M [12,15]	Closed
5	5F8G (EV71), 3OL9 (PV) ^c^	3OL9, 3OLA (chains A/E) [12]	Open
6	5F8N (EV71)	5F8N [15]	Open

^a^ The model was generated using the polymerase and RNA chains from entry 5F8H and a CTP molecule modeled based on the ddCTP molecule from entry 3OLB; ^b^ The model was generated using the polymerase and RNA chains from entry 5F8J with the +1 nucleotide removed and the CTP molecule and the catalytic manganese ions from entry 3BSO; ^c^ The model was generated using the polymerase and RNA chains from entry 5F8G with the last two nucleotides at the 3′-end of the product strand modeled based on entry 3OL9. PV: poliovirus; NV: norovirus.

## Supplementary Materials

The following are available online at www.mdpi.com/1999-4915/10/1/24/s1. Supplementary File S1: NAC.pse (a downloadable PyMOL session file that contains seven structural states S1/S2/S2-3/S3/S4/S5/S6, five states S2m/S2-3m/S3m/S4m/S6m for generating movies, one reference state nvS3, three movies closure1/closure2/translocation, and ten distance labels dist01–dist10 used in the closure1/closure2 movies); Supplementary File S2: NACweb.pse (a web based PyMOL session file that only contains the seven structural states and with all hidden atoms removed to reduce the file size); Supplementary Video S1: Viral RdRP active site closure; Supplementary Video S2: Viral RdRP translocation.
